# Adipose-Derived Mesenchymal Stem Cells Isolated from Patients with Type 2 Diabetes Show Reduced “Stemness” through an Altered Secretome Profile, Impaired Anti-Oxidative Protection, and Mitochondrial Dynamics Deterioration

**DOI:** 10.3390/jcm8060765

**Published:** 2019-05-30

**Authors:** Michalina Alicka, Piotr Major, Michał Wysocki, Krzysztof Marycz

**Affiliations:** 1 Department of Experimental Biology, Wroclaw University of Environmental and Life Sciences, Norwida 27B, 50-365 Wrocław, Poland; michalina.alicka@upwr.edu.pl; 2 2′nd Department of General Surgery, Jagiellonian University Medical College, Kopernika 21, 31-501 Kraków, Poland; piotr.major@uj.edu.pl (P.M.); m.wysocki@doctoral.uj.edu.pl (M.W.); 3 Faculty of Veterinary Medicine, Equine Clinic-Equine Surgery, Justus-Liebig-University, 35392 Gießen, Germany; 4 International Institute of Translational Medicine, Jesionowa, 11, Malin, 55-114 Wisznia Mała, Poland

**Keywords:** adipose-derived mesenchymal stem cells, type 2 diabetes, extracellular microvesicle, insulin resistance, regenerative medicine

## Abstract

The widespread epidemic of obesity and type 2 diabetes (T2D), suggests that both disorders are closely linked. Several pre-clinical and clinical studies have showed that adipose-derived mesenchymal stem cells (ASC) transplantation is efficient and safe. Moreover, scientists have already highlighted the therapeutic capacity of their secretomes. In this study, we used quantitative PCR, a flow cytometry-based system, the ELISA method, spectrophotometry, and confocal and scanning electron microscopy, to compare the differences in proliferation activity, viability, morphology, mitochondrial dynamics, mRNA and miRNA expression, as well as the secretory activity of ASCs derived from two donor groups—non-diabetic and T2D patients. We demonstrated that ASCs from T2D patients showed a reduced viability and a proliferative potential. Moreover, they exhibited mitochondrial dysfunction and senescence phenotype, due to excessive oxidative stress. Significant differences were observed in the expressions of miRNA involved in cell proliferations (miR-16-5p, miR-146a-5p, and miR-145-5p), as well as miRNA and genes responsible for glucose homeostasis and insulin sensitivity (miR-24-3p, 140-3p, miR-17-5p, SIRT1, HIF-1α, LIN28, FOXO1, and TGFβ). We have observed a similar correlation of miR-16-5p, miR-146a-5p, miR-24-3p, 140-3p, miR-17-5p, and miR-145-5p expression in extracellular vesicles fraction. Furthermore, we have shown that ASC_T2D_ exhibited a lower VEGF, adiponectin, and CXCL-12 secretion, but showed an overproduction of leptin. We have shown that type 2 diabetes attenuated crucial functions of ASC, like proliferation, viability, and secretory activity, which highly reduced their therapeutic efficiency.

## 1. Introduction

Over the last decade, type 2 diabetes (T2D) which is recognized as a progressive metabolic disorder, has attained a status of global pandemic, especially in well-developed countries, all over the world. A recent issue of the World Health Organization (WHO) Bulletin reported that 8.5% of the global population suffer from diabetes, and according to the National Institute of Health (NIH), 95% of cases in adult patients are T2D [1,2]. These estimates are also steadily growing. The most significant modifiable risk factor for T2D is severe obesity, thus, a better understanding of the role of adipose tissue in T2D development, is strongly required. A growing body of research has evidenced that dysfunctional adipose tissues, mainly observed during obesity, are characterized by impaired insulin signaling, insulin resistance, adipocyte hypertrophy, and macrophage infiltration [3]. T2D is a leading cause of cardiovascular diseases, lower limb amputations, kidneys failure, as well as retinopathy, which can directly result in blindness [2].

Adipose tissue becomes an easily accessible source of the unique population of adipose tissue-derived mesenchymal stem cells (ASCs), characterized by their fibroblast-like morphology, colony forming properties [4], capacity for self-renewal and a considerable proliferative rate, resulting in the overexpression of Ki-67, which is widely used as a proliferation marker in clinics [5]. Moreover, they possess a unique ability to differentiate into mesenchymal lineage cells, such as chondrocytes, osteoblasts, and adipocytes, under proper conditions, which makes them an attractive tool for tissue engineering and regenerative medical applications [6]. The isolation of ASCs from subcutaneous adipose tissue is considered to be a relatively easy procedure, providing a high quantity of mesenchymal stem cells (MSCs), compared to the inter alia bone-marrow-derived stem cells. Unfortunately, many stem cell properties can be sorely influenced by some factors, such as ageing and metabolic disorders. Several studies have shown, that MSCs isolated from aged individuals exhibit a reduced proliferative activity and osteogenic potential, as well as senescent features, resulting in more senescence-associated β-galactosidase-positive cells, leading to a decreased cell viability, in comparison to young donors [7].

Moreover, ASCs have been shown to exhibit immunomodulatory properties, which make them promising therapeutic agents, since T2D is linked to chronic inflammation [8,9]. While increasing evidence has shown that the unique properties of ASCs are explained by their ability to synthesize and secrete a broad range of different growth factors, including inter alia, bone morphogenetic protein 2 and 4 (BMP 2 and 4), leptin, adiponectin, stromal cell-derived factor -1α (SDF-1α), and the vascular endothelial growth factor (VEGF), extracellular microvesicles (MVs) released by ASCs have gained more interest for their paracrine communication capacity in the field of ASC transplantation therapy [10,11,12]

Moreover, MVs have been observed to carry and release not only proteins but also nucleic acids, like mRNAs and non-coding small regulatory RNAs, in particular microRNAs (miRNA), which have the ability to cause pleiotropic effects on target cells [13]. miRNA, including miR-17-5p, miR-24-3p, and miR-145-5p, play a substantial role in the pathogenesis of diabetes mellitus affecting the functioning of the β-cell, and proliferative activity, insulin resistance, and glucose homeostasis, mainly through the Phosphoinositide 3-Kinase (PI3K)/AKT Serine/Threonine Kinase (AKT) pathway [14,15]. Recent studies have shown that multiple signaling pathways, including Wnt, BMP, FGF, and in particular miRNAs, which are involved in the pro-regenerative capacity of ASCs, are seriously altered in diabetic patients [16,17].

Excessive oxidative stress has been proposed as one of the major factor leading to insulin resistance, impairment of glucose tolerance, and β-cell dysfunction [18,19]. Overproduction of reactive oxygen species (ROS), such as superoxide (O^–^_2-_, nitric oxide (NO), and hydrogen peroxidase (H_2_O_2_), with a simultaneous reduction of antioxidant defenses from the superoxide dismutase activity (SOD), alters the cellular homeostasis and induces apoptosis [19]. The prolonged generation and accumulation of ROS or NO might affect many cellular dysfunctions, including the induction of a senescence character in the ASCs, a decreased ability to synthesize, secretion of particular growth factors, loss of immunomodulatory effects, and finally result in cell death through the necrotic or apoptotic mechanisms [20,21]. The most important sources of ROS production are the mitochondrial electron-transport system, NAPDH oxidase, and the cytochrome P450 [21]. Moreover, apart from the altered energy and overproduction of ROS, mitochondrial dysfunction is highly linked to an impaired cellular calcium (Ca^2+^) homeostasis, which causes ER stress, and leads to the activation of a signaling network called unfolded protein response (UPR) [22,23]. To maintain their health, the mitochondria undergo coordinated cycles of fusion (the joining of two organelles into one) and fission (the division of one mitochondrion into two daughter mitochondria). Moreover, mitochondrial movements through the cytoskeleton and mitophagy (targeted destruction via autophagy) have also been observed [24]. Several studies have shown that insulin resistance leads to an imbalance in the mitochondrial fission/fusion ratio, manifested by a higher fission activity, leading to an inefficient mitochondrial network [25]. In other words, the metabolic disorders impair the subsequent modification of mitochondrial dynamics, resulting in mitochondrial dysfunction.

According to the latest data, ASCs from aged donors [26,27], as well as from individuals suffering from metabolic disorders [28,29,30] showed a diminished regenerative potential. In the present study, we used a human model for type 2 diabetes, the most common form of diabetes that is frequently associated with obesity and metabolic alterations in adipose tissue. We examined the effect of diabetes on the morphology, proliferative activity, and viability of ASCs isolated from subcutaneous adipose tissue. Moreover, we focused on the cellular senescence, the impairment of anti-oxidative defenses, mitochondrial dynamics deterioration, the miRNA expression profile, and secretory activity. We believe that this approach might offer a worthwhile path to explain the effect of type 2 diabetes on the regenerative potential of ASCs.

## 2. Experimental Section

All reagents used in the study were purchased from Sigma-Aldrich (St. Louis, MO, USA), unless otherwise stated.

The study was approved by the independent ethics committee of the Jagiellonian University, Krakow, Poland (1072.6120.220.2018). Informed consent for the surgical treatment was obtained from all patients, before the procedure.

### 2.1. Isolation and Culture of Human Adipose-Derived Mesenchymal Stem Cells (hASCs)

Human adipose-derived mesenchymal stem cells were isolated from subcutaneous adipose tissues (AT), from non-diabetic (ASC_CTRL_) donors (both sexes, age 38–56; BMI = 15.45 ± 2.21; *n* = 6) or patients with clinically confirmed type 2 diabetes (ASC_T2D_) (both sexes, age 36–69; BMI = 41.50 ± 5.50; *n* = 6) with no history of smoking.

Human AT was collected during the surgical procedure of total hip arthroplasty. Immediately after the tissue collection, the specimens were transferred to a tissue-transport medium, consisting of Hank’s Balanced Salt Solution (HBSS), supplemented with 1% antibiotic–antimycotic solution (Penicillin/Streptomycin/Amphotericin B, P/S/A). Immediately upon arrival, the adipose tissue pieces were washed with phosphate-buffered saline (PBS), supplemented with 1% P/S, mechanically disintegrated, and treated with 1 mg/mL solution of type I collagenase, at 37 °C, for 40 min. After incubation, the digested tissue was centrifuged (1200× *g*, 10 min) to obtain a cell pellet. Then, the supernatant was discarded and the cell pellet was resuspended in PBS, and centrifuged again (300× *g*, 4 min). After the second centrifugation, cells were seeded in a T25 cm^2^ polystyrene culture flask, and cultured in Dulbecco’s Modified Eagle Medium (DMEM), with nutrient F-12 Ham supplemented with 10% of Fetal Bovine Serum (FBS), 1% Penicillin/Streptomycin/Amphotericin B (P/S/A) and 0.5% of Gentamicin, upon reaching approximately 85% confluence. Then, the cells were gently recovered using TrypLE Express solution (Life Technologies, Carlsbad, CA, USA) and re-seeded in a T75 cm^2^ flask. The medium was renewed every 2–3 days. The cells were passaged, three times, prior to the experiment.

### 2.2. Proliferation Rate Assay

Cell proliferative activity was determined in two ways—using the reazurin-based assay kit (TOX -8) for population doubling-time (PDT) evaluation, and with the BrdU Cell Proliferation ELISA Kit (Abcam, Cambridge, UK). For the assessment of the PDT value, thrice passaged ASCs were seeded onto 24-well plates, at a density of 2 × 10^4^ cells per well. After, 24, 48, 72, and 96 h of culture, media were replaced with a 10% resazurin dye solution, prepared in a fresh complete culture medium. After 2 h of incubation at 37 °C in a CO_2_ incubator, the supernatants were subsequently transferred to a 96-well plate and the absorbance levels were measured, spectrophotometrically (Epoch, Biotek, Bad Friedrichshall, Germany), with the wavelengths of 600 nm and 690 nm as the reference length. The PDT value was calculated with the support of online software [31].

For the cell proliferative activity determination using the BrdU Cell Proliferation ELISA Kit, the cells were seeded onto 96-well plates, at a density of 6 × 10^3^ cell/well in 100 µL of culture media. The procedure was performed following the instructions provided by the supplier. After 24 h, 20 µL of the diluted BrdU reagent was added directly to the culture medium and incubated for 18 h, at 37 °C, in standard growth conditions. Then, the cells were fixed, permeabilized, and incubated with anti-BrdU monoclonal antibody detector. After 1 h of incubation, horseradish peroxidase-conjugated goat anti-mouse antibody was added (incubation—30 min, RT). After washing and incubation with the 3,3’,5,5’-tetramethylbenzidine (TMB) chromogenic substrates, the colored reaction product was quantified by spectrophotometric measurement (Epoch, Biotek, Bad Friedrichshall, Germany), at a wavelength of 450 nm.

### 2.3. Extracellular Oxidative Stress Evaluation

Extracellular oxidative stress level quantification was performed using two methods. In order to assess the total superoxide dismutase (SOD) activity and the nitric oxide (NO) concentration, ASCs were seeded in 24-well plates, at a concentration of 20 × 10^4^ cells per well. After three days of culture, the growth medium was replaced with a phenol-red-free medium. On the fifth day of the experiment, the cell culture supernatants were collected and the SOD activity was determined using a commercially available SOD Assay Kit, while NO accumulation was measured with a Griess Reagent Kit (Life Technologies, Carlsbad, CA, USA). All assays were performed according to the manufacturer’s protocols.

### 2.4. Determination of Cellular Senescence

In order to evaluate cellular senescence, the lysosomal senescence-associated β-galactosidase enzyme (SA-β-gal) activity was determined using the Senescence Cells Histochemical Staining Kit. The procedure was carried out in accordance to the manufacturer’s instructions. After 3 days of propagation, the cells were fixed with the provided formaldehyde-based fixation buffer (6 min, RT), then washed with PBS and stained with an X-gal solution containing 5-bromo-4-chloro-3-indolyl-b-D-galactopyranoside and warmed at 37 °C. The incubation was carried out overnight at 37 °C, then the supernatants were collected and spectrophotometrically measured (Epoch, Biotek, Bad Friedrichshall, Germany) at 450 nm. Moreover, blue-stained senescent cells were visualized and imaged under inverted microscope.

### 2.5. Cell Morphology Evaluation (SEM Analysis)

Detailed cell morphology was evaluated using Scanning Electron Microscopy. To carry out the SEM analysis, the cells were fixed with 4% paraformaldehyde (PFA), washed with distilled water, and dehydrated in a graded ethanol series. The dehydrated samples were sprinkled with gold (ScanCoat 6, Oxford, UK) and transferred to the microscope chamber. The observation was performed using an SE1 detector, at 10 kV of filament tension.

### 2.6. Fluorescent Microscopy

Visualization of the F-actin filaments was performed using Phalloidin Atto 590 staining. Cells were fixed with 4% PFA for 45 min, at RT. Afterwards, the cells were washed with PBS and permeabilized using 0.3% Tween 20 in PBS, for 15 min. Then, the cells were incubated with Phalloidin Atto 590 (1:800 dilution) in PBS, for 45 min, in the dark.

Mitochondria were stained using MitoRed dye. On the last day of osteogenic differentiation, the cells were incubated with MitoRed solution (dilution 1:1000) at 37 °C. After 30 min of incubation, the cells were fixed with 4% PFA and rinsed, three times, with PBS. The cells were counterstained with 4′,6-diamidino-2-phenylindole (DAPI), in order to visualize the cells’ nuclei. Moreover, the mitochondrial membrane potential was determined using JC-1 staining. The cells were incubated with JC-1 dye (1:100 dilution), for 30 min at 37 °C, in a CO_2_ incubator, rinsed with PBS, and fixed using 4% PFA, and then counterstained with DAPI.

Cell viability was assessed using Ki-67 nuclear antigen staining. Cells were fixed with 4% PFA for 45 min at RT, washed three times with PBS and permeabilized with 0.5% Triton X-100 for 10 min, at RT. After incubation, the cells were rinsed three times with PBS and incubated with 1% Bovine Serum Albumin (BSA) and 22.52 mg/mL glycine solution, in Phosphate Buffered Saline with 0.2 % Tween 20 (PBST), for 30 min, to avoid unspecific binding of the antibody. Then, the cells were incubated with the primary anti-Ki-67 antibody (dilution 1:100 in 1% BSA in PBST solution) (Abcam, Cambridge, UK), overnight, at 4 °C. Thereafter, the cells were rinsed with PBS and incubated with secondary Alexa Fluor 488-conjugated anti-rabbit antibody (1:1000) (Abcam, Cambridge UK) for 1 h, at RT, in the dark. Excess secondary antibody was washed off and the cells were counterstained with DAPI. All observations were performed and documentation was made using a confocal microscope (Observer Z1 Confocal Spinning Disc V.2 Zeiss with live imaging chamber). Pictures were analyzed using the ImageJ software.

### 2.7. Quantitative Cell Analysis Using a Flow Cytometry-Based System

Intracellular Reactive Oxygen Species (ROS) were detected using a Muse® Oxidative Stress Kit, based on dihydroethidium (DHE). The assay allowed to distinguish two populations of cells—ROS (−) live cells and ROS (+) cells exhibiting high content of ROS.

Changes in the mitochondrial membrane potential were evaluated using the Muse® Mitopotential Assay Kit, which evaluated the percentage of the four cell populations—live, depolarized/live, depolarized/dead, and dead cells.

The apoptosis rate was assessed with the Muse® Caspase-3/7 Kit and Muse® Annexin V & Dead Cell Assay Kit. Both assays were used to determine the percentage of live, early and late apoptotic, as well as dead cells. All procedures were performed according to the protocols provided by the supplier and the results were acquired by means of a Muse Cell Analyzer (Merck, Darmstad, Germany).

### 2.8. ELISA Assay (VEGF, CXCL12/SDF-1, Adiponectin, and Leptin)

Extracellular concentrations of the Vascular Endothelial Growth Factor (VEGF), Stromal Cell-Derived Factor 1 (SDF-1), Adiponectin, and Leptin were determined in a cell-free, conditioned, culture medium, collected from the investigated cells. When the cells reached 85% of confluence, the culture supernatants were replaced with a fresh medium, which was subsequently conditioned for the next 48 h. The secreted proteins were detected using the Quantitative ELISA kits purchased from the R&D Systems (Minneapolis, MN, USA), according to the manufacturer’s instructions.

### 2.9. Analysis of mRNA and microRNA Expression (RT-qPCR)

For analysis of mRNA and miRNA expression in ASCs, thrice passaged cells pellets were lysed in TRI Reagent® Solution. In contrast, in order to obtain miRNA from MVs, the cells were cultured in a serum-free medium for 24 h. Then, the medium was collected, centrifuged at 2000× *g* for 10 min at 4 °C, to remove cell debris, and then concentrated to 100 µL, using Amicon Ultra-15 Centrifugal Filter (3000 MWCO, Merck, Darmstad, Germany).) via centrifugation (4000× *g* at 4 °C).

Total RNA was isolated using Chomczyński’ method [32]. Total RNA was quantified and the 260/280 nm absorbance ratios were determined using a nanospectrophotometer (Epoch, Biotek, Bad Friedrichshall, Germany). For the mRNA analysis, gDNA was digested using DNase I, RNase-free (Thermo Scientific^TM^, Waltham, MA USA), and cDNA was synthesized using a RevertAid RT Reverse Transcription Kit (Thermo Scientific^TM^, Waltham, MA, USA). A total of 150 ng of the total RNA, served as a template for a single reaction. The procedures were performed, following the protocol provided by the producer. qRT-PCR was performed on a CFX Connect^TM^ Real-Time PCR Detection System (Bio-Rad, Hercules, CA, USA). The PCRs were performed in 10-µL reaction volume, using 1 µL of cDNA, 500 nM of specific primers, using the SensiFast SYBR and Fluorescein Kit (Bioline, Cincinnati, OH, USA). The sequences of the specific primers are shown in Table 1. The quantitative expression of the investigated genes was calculated by the 2^−ΔΔCT^ method, relative to the housekeeping gene (GAPDH).

In order to determine the microRNA expression, 500 ng of each RNA sample was polyadenylated, and cDNA synthesis was performed using the Mir-X miRNA First-Strand Synthesis Kit (Clontech Laboratories, Incorporated, Mountain View, CA, USA), in accordance with the manufacturers’ instructions. The obtained cDNA was used for qRT-PCR (total volume 25 µL) with the specific primers listed in Table 2 and SYBR Advantage qPCR Premix (Clontech Laboratories, Incorporated, Mountain View, CA, USA) in CFX Connect^TM^ Real-Time PCR Detection System (Bio-Rad, USA). The relative miRNA expression level was calculated using the 2^−ΔΔCT^ algorithm, in relation to U6 snRNA, used as an endogenous control.

### 2.10. Statistical Analysis

Statistical analysis was performed using unpaired Student’s *t*-test with the GraphPad Prism 5 software (San Diego, CA, USA). In the study, we compared the non-diabetic (*n* = 6) and diabetic patient (*n* = 6) groups. Asterisk (*) indicated statistical significance between the two groups. A *p* value less than 0.05 (*p* < 0.05) was summarized with one asterisk (*), one less than 0.01 (*p* < 0.01) with two asterisks (**), and one less than 0.001 (*p* < 0.001) with three asterisks (***).

## 3. Results and Discussion

### 3.1. Type 2 Diabetes Diminishes ASC Proliferation and Increases Apoptosis

**Figure 1:**
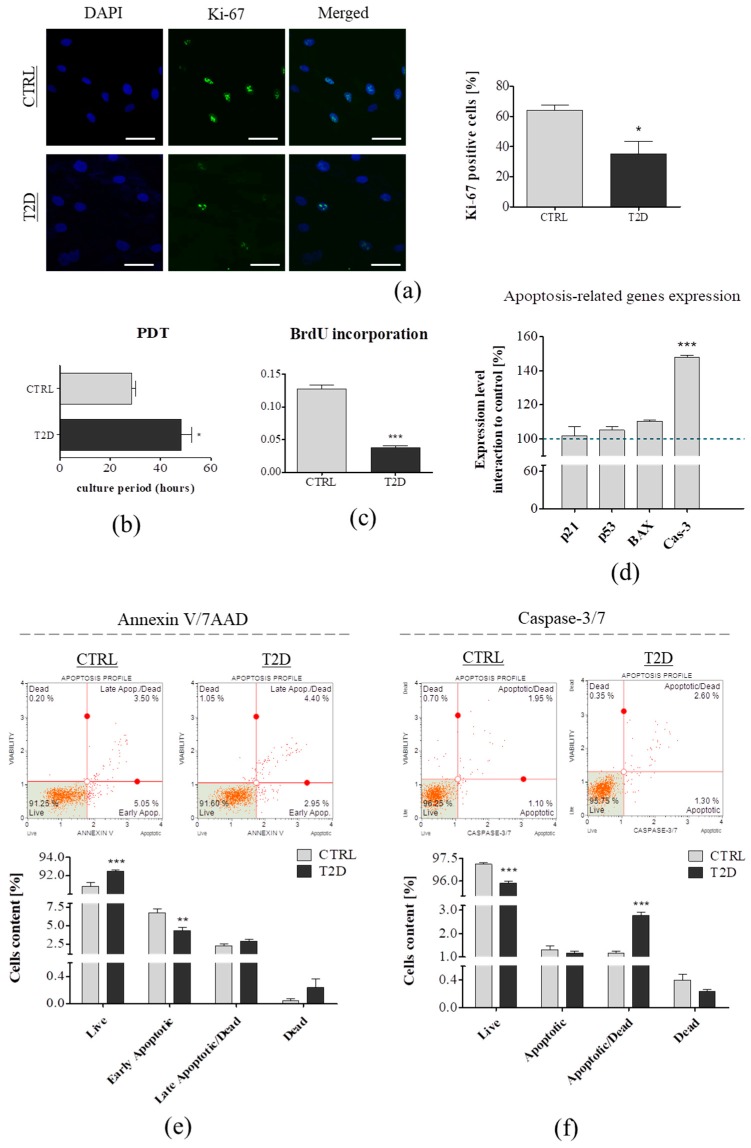
Comparison of proliferation and apoptosis in adipose-derived mesenchymal stem cells from non-diabetic donors (ASC_CTRL_) (*n* = 6) and patients with clinically confirmed type 2 diabetes (ASC_T2D_) (*n* = 6). (**a**) Representative images of immunofluorescence staining for Ki-67 (green). Nuclei were counterstained with 4′,6-diamidino-2-phenylindole (DAPI) (blue). Ki-67 expression in ASCs was showed as Ki-67 positive cells percentage. Scale bar: 50 µm. (**b**) population doubling-time (PDT) value was determined using TOX-8 Resazurin-based Assay. (**c**) BrdU incorporation assay. Cells were cultured with BrdU labeling medium for 18 h. (**d**) Transcript levels of the apoptosis-related genes was determined using the qRT-PCR method. The black dotted line indicated the transcript levels of genes in the ASC_CTRL_ group. (**e**,**f**) Evaluation of apoptosis and cell death using the Muse Cell Analyzer. The percentage of live, apoptotic, apoptotic/dead, and dead cell was assessed using thee Annexin/7AAD and Caspase-3/7 tests. Results are expressed as mean ± SD. Statistical significance is indicated by asterisks (*); * *p* < 0.05, ** *p* < 0.01, *** *p* < 0.001, as examined by unpaired Student’s *t*-test.

ASCs exhibited an extensive capacity for proliferation, self-renewal, and showed the potential to differentiate into insulin—producing cells, which make them a good candidate for cell therapy applications in many pathologies, including type 2 diabetes. Therefore, it was not surprising that extensive studies were necessary to derive which type of ASC transplantation would be the best—autologous or allogenic. Numerous studies have reported that ASC stemness can be diminished by the ageing of donors or metabolic disorders, including insulin resistance, hyperglycemia, and lipid accumulation, leading to T2D. To compare the cellular proliferation of ASCs derived from diabetic and non-diabetic patients, we performed Ki-67 expression analysis, using immunofluorescence staining, PDT calculation, and BrdU Cell Proliferation Assay. Ki-67 is a nuclear protein highly associated with cell proliferation, and is expressed in the G1, S, G2, and M cell cycle phases, but is not present in the G0 phase. The Ki-67 percentage score was acquired as the percentage of positively-stained cells, among the total number of ASC cells assessed [33,34]. We noted that, ASC_CTRL_ displayed a higher expression of Ki-67 (*p* < 0.05). More than 60% of the control cells were Ki-67-positive, while the percentage of ASC_T2D_ expressing Ki-67 was approximately 40% (Figure 1A). Differences in the proliferation rate of ASCs from the CTRL and T2D groups were observed in the in vitro culture, using resazurin-based TOX-8 assay (Figure 1B)—to estimate the PDT—and the BrdU Cell Proliferation Assay Kit (Figure 1C). ASCs derived from the non-diabetic donors had a significantly shorter population double-time, compared to the ASC_T2D_ (*p* < 0.05) (Figure 1B). BrdU assay detected the incorporated 5-bromo-2′deoxyuridine (BrdU) (pyrimidine analog), in the newly synthesized DNA of the proliferating cells. We observed a two-fold lower content of the incorporated BrdU, in the DNA of ASCs from diabetic patients (*p* < 0.001) (Figure 1C). The decreased Ki-67 expression, significantly higher PDT, and lower content of incorporated BrdU in DNA, indicated a reduced ASC_T2D_ proliferation activity. Similarly, many recent studies on the animal models have demonstrated that several factors, such as aging and metabolic diseases, strongly affect ASCs proliferation [29,35,36]. Moreover, it has been demonstrated that reduced proliferation activity was closely related to telomere length shortening in patients with age-related diseases and type 2 diabetes [26,37].

Cell apoptosis was estimated by quantitative polymerase chain reaction and Muse Cell Analyzer. Thus, we compared the basal transcript levels of the genes encoding the proteins related to apoptosis (p21, p53, BAX, Cas-3) in ASCs derived from diabetic and non-diabetic patients. Overall, p21, p53, and BAX expression was higher in the T2D group. Moreover, we noted an overexpression of Cas-3 (*p* < 0.001) in the ASCs from diabetic donors (Figure 1D). Annexin V and Dead Cell analysis allowed for the quantitative analysis of live (Annexin V (−) and 7AAD (−)), early apoptotic (Annexin V (+) and 7AAD (−)), late apoptotic (Annexin V (+) and 7AAD (+)), and necrotic cells (Annexin V (−) and 7AAD (+)). We observed a significantly higher content of living cells in the T2D group (*p* > 0.001), whereas fewer cells from the T2D group exhibited an early apoptotic stage (*p* > 0.01). Interestingly, there were more late apoptotic and dead cells in the T2D group (Figure 1E). Further examination of cellular apoptosis by the Muse Cell Analyzer showed a significantly lower content of live cells in the ASC population derived from diabetic donors (*p* > 0.001), and a greater percentage of apoptotic/dead cells (*p* > 0.001) (Figure 1F). The obtained results correlated with our previous study on animal model, which showed that the metabolic syndrome enhanced the apoptosis in ASCs [12,29,35].

### 3.2. Cellular Senescence and Extracellular Oxidative Stress in ASCs are Associated with Type 2 Diabetes

**Figure 2:**
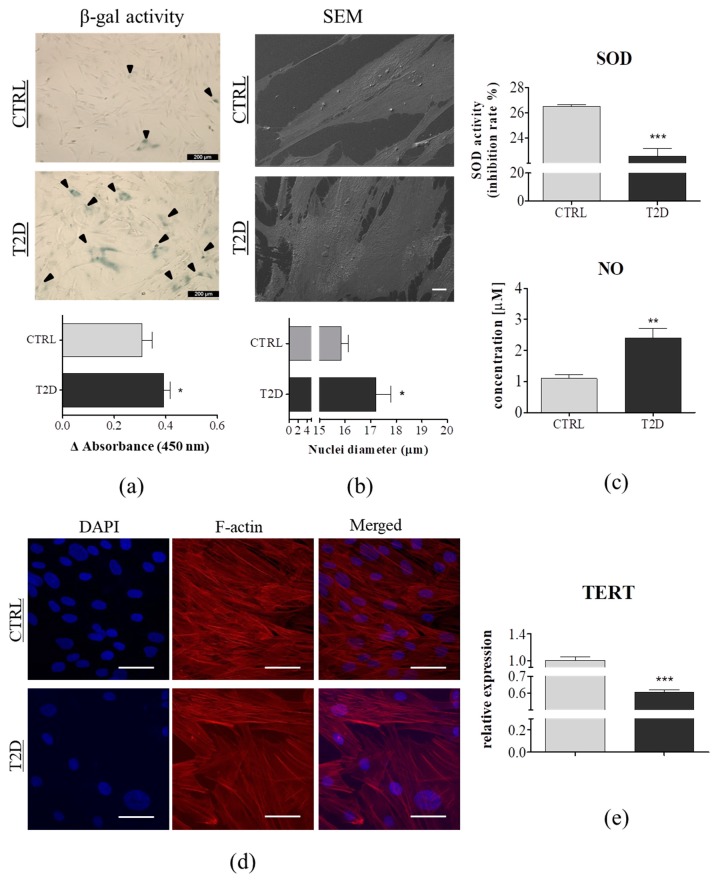
Changes in cellular senescence and extracellular oxidative stress in ASC_CTRL_ (*n* = 6) and ASC_T2D_ (*n* = 6). (**a**) Representative images of senescence-associated β-galactosidase (SA β-gal) staining in ASC_CTRL_ and ASC_T2D_. Positive blue staining of SA β-gal appeared in the senescent T2D group; indicated by black arrows. SA β-gal staining was quantified using spectrophotometry (450 nm); showed as mean of Δ Absorbance. Scale bar: 200 µm. (**b**) Visualization of ASCs morphology using SEM. Mean diameter of the cell nuclei measured on the basis of SEM images. Scale bar: 20 µm, magnification: 1000×. (**c**) Quantification of extracellular oxidative stress factors. The levels of secretory superoxide dismutase (SOD) showed as mean of SOD activity (inhibition rate [%]) and released nitric oxide (NO) was determined in cell-free culture supernatants. (**d**) Representative pictures of F-actin visualization with Phalloidin Atto-594 staining, using confocal microscopy. Scale bar: 50 µm. (**e**) Relative expression of telomerase reverse transcriptase (TERT) in ASC_CTRL_ and ASC_T2D_ qRT-PCR. Results expressed as mean ± SD. Statistical significance indicated as asterisk (*): * *p* < 0.05, ** *p* < 0.01, and *** *p* < 0.001 were examined by an unpaired Student’s *t*-test.

Senescent cells are characterized by an increased activity of senescence-associated β-galactosidase (SA β-gal) [38]. Using the SA β-gal assay (SA β-gal positive blue-green stained cells), we compared cellular senescence in ASCs derived from non-diabetic and diabetic patients. We observed an increased percentage of SA β-gal positive cells in ASC_T2D_ (black arrows). The result was confirmed by the spectrophotometric measurement; the dye absorption was significantly higher in the ASCs derived from the diabetic patients (*p* < 0.05) (Figure 2A). Moreover, we observed a significant downregulation of the telomerase reverse transcriptase (TERT) in ASC_T2D_ (*p* < 0.001) (Figure 2E). Telomerase, a complex of the reverse transcriptase protein and a template RNA TERC (telomerase RNA component) is responsible for the synthesis of telomeric repeats onto the chromosomes ends. The mechanism protects cells from the replication-dependent loss of telomere, which leads to cellular senescence in highly proliferative cells, such as ASCs [39]. It has been previously reported that, telomerase shortening induces cellular senescence and apoptosis, and is highly associated with the TERT activity [40]. Its downregulation leads to telomere shortening, resulting in cellular senescence and apoptosis.

Morphological alterations were evaluated using SEM imaging (Figure 2B), f-actin, and nuclei staining (Figure 2D). ASCs derived from the non-diabetic patients exhibited uniform and bipolar fibroblastic-like phenotype, whereas a great majority of ASC_T2D_ were rather flat, and had irregular cells with enlarged nuclei. Moreover, determination of nuclear size, using SEM, revealed that the nuclei of ASCs derived from the T2D-patients group, showed a greater diameter than the control group (*p* < 0.05) (Figure 2B). According to several studies, the enlarged cell nuclei is a biomarker of cellular senescence [41,42]. Interestingly, the phalloidin and DAPI staining showed that, both, the smaller ASC_CTRL_ and the larger ASC_T2D_, exhibited a well-developed cytoskeleton (Figure 2D). Our data were partially consistent with our previous study, in which we demonstrated a similar morphology of ASCs derived from horses suffering from equine metabolic syndrome (EMS) [29]. The obtained results indicated that ASCs derived from type 2 diabetes donors exhibited a phenotype of senescent cells.

Cellular senescence can be induced by various factors, including oxidative stress [43]. SOD enzymes control the levels of NO and ROS, thus, protecting the cells from potential damage caused by excessive ROS. In order to assess the extracellular oxidative stress, SOD and NO assays were performed using cells-free supernatants collected from ASCs, after five days of culture. We observed a significantly lower total SOD activity in the T2D group, represented by the percentage of inhibition (*p* < 0.001) and a higher concentration of NO (*p* < 0.01) (Figure 2C). Excessive release of NO, and inhibition of the SOD activity indicated an elevated oxidative stress level in ASCs derived from diabetic donors. Our research stayed in a good agreement with the findings observed in both human and animal models [28,44,45]. As reported by Lee et al., the oxidative stress in adipose tissue can cause an increase in preadipocyte proliferation, adipogenesis, and the size of mature adipocytes, and therefore, increases fat mass [46].

### 3.3. Comparison of the Mitochondrial Dynamics and Mitochondrial Membrane Potential

**Figure 3:**
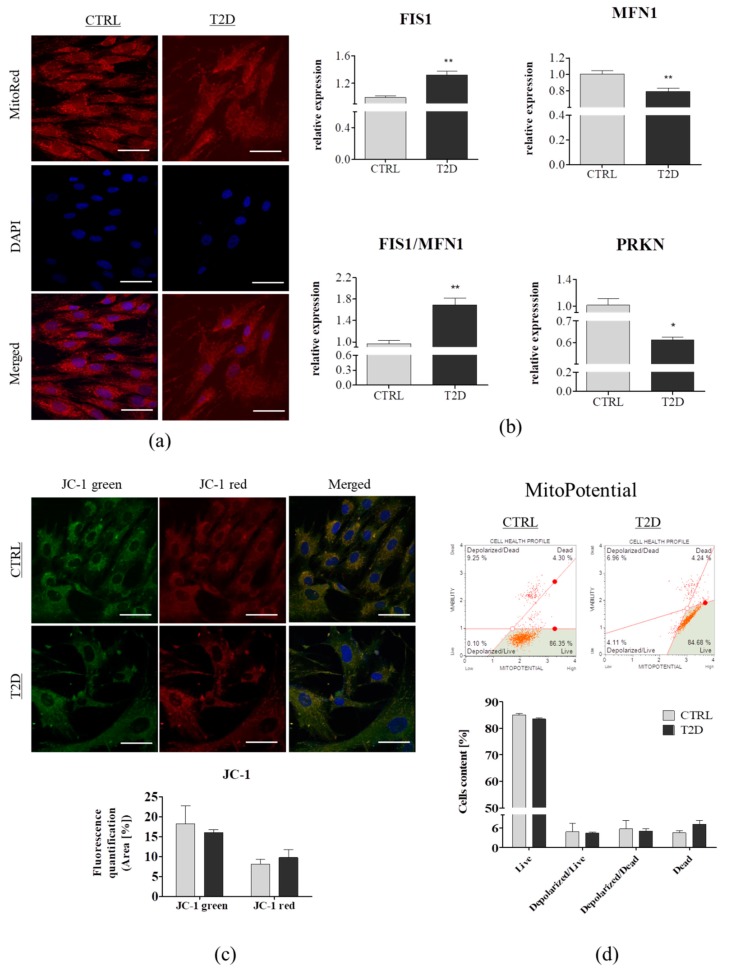
Mitochondrial dynamics and mitochondrial membrane potential in the ASC_CTRL_ (*n* = 6) and ASC_T2D_ (*n* = 6). (**a**) Representative images of mitochondria visualization in ASCs with MitoRed (red) and DAPI (blue) staining, using confocal microscopy. Scale bar: 50 µm. (**b**) Transcript levels of mitochondrial dynamics-related genes, assessed using qRT-PCR. (**c**) Mitochondrial membrane potential estimation by JC-1 staining, using confocal microscopy. (**d**) MitoPotential Analysis using the Muse Cell Analyzer. Fluorescence intensity of JC-1 staining was quantified using Image J. Results are expressed as mean ± SD. Statistical significance is indicated by asterisks (*): * *p* < 0.05, ** *p* < 0.01 were examined by an unpaired Student’s *t*-test. Scale bar: 50 µm.

Mitochondria are extremely dynamic organelles, undergoing frequented cycles of fusion and fission, which serve to regulate mitochondrial functioning, by enabling their transient and rapid adaptations to the metabolic requirements of cells [47]. To assess the mitochondrial dynamics in ASCs derived from a diabetic donor, we performed mitochondria staining with MitoRed (Figure 3A) and quantitative real-time PCR (Figure 3B). Mitochondria in the ASCs from control group, exhibited a typical tubular structure, which formed a robust mitochondria network. By contrast, the ASC_T2D_ contained round mitochondria characterized by a fragmented phenotype (Figure 3A). Mitochondrial network fragmentation indicated diminishing fusion or escalating fission, which was confirmed by qRT-PCR. Overexpression of FIS1 in ASC derived from diabetic donors, led to mitochondria fragmentation (*p* > 0.01), whereas, the transcript level of MFN1 responsible for the regulation of mitochondrial fusion was significantly decreased in these cells (*p* > 0.01). Overexpression of FIS1 led to an increase of the FIS1/MFN1 ratio (*p* > 0.01). Moreover, PRKN (mitophagy stimulator) expression was significantly reduced in ASC_T2D_ (*p* > 0.01) (Figure 3B). Taken together, these results indicated that fission appeared to be predominant over fusion, in these cells. Moreover, downregulation of PRKN expression is known to impair mitophagy, which is crucial for the clearance of damaged mitochondria. This effect was consistent with our previous studies, where we observed an increased mitochondrial fission in ASC derived from EMS horses [28,35,45]. Moreover, we have demonstrated that mitochondrial fragmentation is linked to an increased release of NO and impaired SOD activity in ASC_T2D_. These results showed that the metabolic disorders affected the mitochondrial network and probably induced their dysfunction on its own, not only by enhancing mitochondrial fission but also by impairing mitochondrial autophagy. As is well-known, dysfunctional mitochondria are the main source of ROS, therefore, the removal of damaged mitochondria via mitophagy is important for the maintenance of oxygen homeostasis, by a lower ROS generation. Experimental studies have previously shown that under normal conditions, fission is a process that separates damaged mitochondria from the network, and permits their removal, through mitophagy [48], which is impaired during the metabolic syndrome. Furthermore, present results stay in good agreement with our previous findings [29], as well as the results of Wang et al. [49], which showed that mitochondria impairment is highly associated with apoptosis, which is preceded by mitochondrial depolarization. JC-1 staining is widely used to monitor mitochondrial health in a variety of cell types. Thus, mitochondria depolarization is indicated by a decrease in red (~590 nm)/green (~529 nm) fluorescence intensity ratio. ASC_T2D_ exhibited no alternation in JC-1 green/red fluorescence intensity (Figure 3C). MitoPotential test using the Muse Cell Analyzer revealed higher levels of dead cells in the T2D group, whereas, there were no significant differences in mitochondrial membrane depolarization between the tested groups (Figure 3D). These results suggest that metabolic alternations led to mitochondrial dysfunction, and consequently, induced cell death via apoptosis.

### 3.4. Expression of mRNA and miRNA Associated with Cell Proliferation and Insulin Resistance

**Figure 4:**
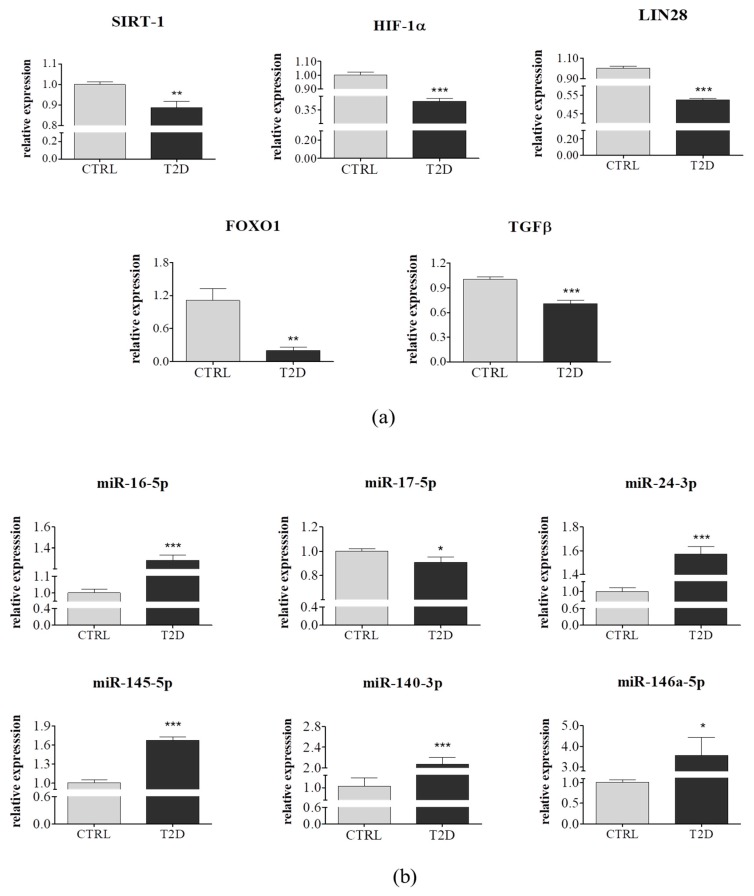
Transcripts levels of selected mRNA and miRNA in ASCs derived from a control group (*n* = 6) and from type 2 diabetic individuals (*n* = 6). (**a**,**b**) Both, mRNA and miRNA expression profiles were determined using quantitative PCR. Results are expressed as mean ± SD. Statistical significance are indicated by asterisks (*): * *p* < 0.05, ** *p* < 0.01, and *** *p* < 0.001 were examined by unpaired Student’s *t*-test.

In order to evaluate, both, the mRNA and miRNA expression patterns of the selected genes and the miRNA involved in cellular proliferation, the regulation of glucose homeostasis, and insulin sensitivity, qRT-PCR was performed. We observed that the expression level of genes involved in the control of the glucose–lipid metabolism, namely, SIRT-1, LIN28, and FOXO1 were highly downregulated in ASCs derived from diabetic patients (*p* < 0.01, *p* < 0.001, and *p* < 0.01, respectively). Similarly, TGFβ and HIF-1α transcript levels appeared to be significantly higher in the control group (*p* < 0.001) (Figure 4A). Several reports have shown that SIRT-1 is an important regulator of glucose metabolism, insulin sensitivity [50], and ASC differentiation towards osteoblasts and chondrocytes, in vitro [51]. Our result was in a good agreement with several animal studies that have provided strong evidence on the positive effect of decreased SIRT-1 activity, on the development of T2D-related conditions, including insulin resistance and obesity. Furthermore, SIRT-1 was a crucial regulator of FOXO-mediated transcription, during the development of insulin resistance [51]. A growing body of evidence indicates a dual role of HIF-1α, in insulin resistance. Overexpression of HIF-1α promotes cell survival via repression of p53, phosphorylated-p53, and p21, under normoxia [52], on the other hand, inhibition of HIF-1α activity improves insulin sensitivity [53]. Our results suggest that metabolic disorders diminish cell viability and increase cell apoptosis, through downregulation of HIF-1α. As expected, the expression of Lin28 was significantly reduced in ASC_T2D_, suggesting that these cells lost their self-renewal abilities, at least partially, whereas a diminished expression of TGFβ might indicate impaired immunomodulatory properties.

The miRNA expression was analyzed for the following miRNAs: miR-16-5p, 17-5p, 24-3p, 145-5p, 140-3p, and miR-146. ASCs derived from diabetic donors were characterized by increased expression of miRNA involved in cell proliferation, such as miR-16-5p miR-146a-5p and miR-145-5p (*p* < 0.001), which when overexpressed, suppressed proliferation [54]. Furthermore, some of the studied miRNAs played crucial roles in the glucose and lipid metabolism, like miR-24-3p (*p* < 0.001), which mainly contributed to the increase of glucose uptake [55] and miR-17-5p, whose expression was highly reduced in ASC_T2D_ (*p* < 0.05), this strongly correlated with the findings revealed by Dong et al., who showed that miR-17 expression was downregulated, in a high glucose condition [56]. Moreover, we observed an upregulation of miR-140-3p, which was highly linked to T2D (*p* < 0.001) (Figure 4B). These results indicated that type 2 diabetes significantly altered, both, the mRNA and miRNA expression profiles of ASC, thus, reducing their viability, proliferation activity, glucose homeostasis, and insulin sensitivity.

### 3.5. Alternations in Selected miRNA and Protein Levels in ASCs Derived from T2D Patients Exhibit their Impairment of Proliferation Activity, Regenerative Potential, and Insulin Sensitivity

**Figure 5:**
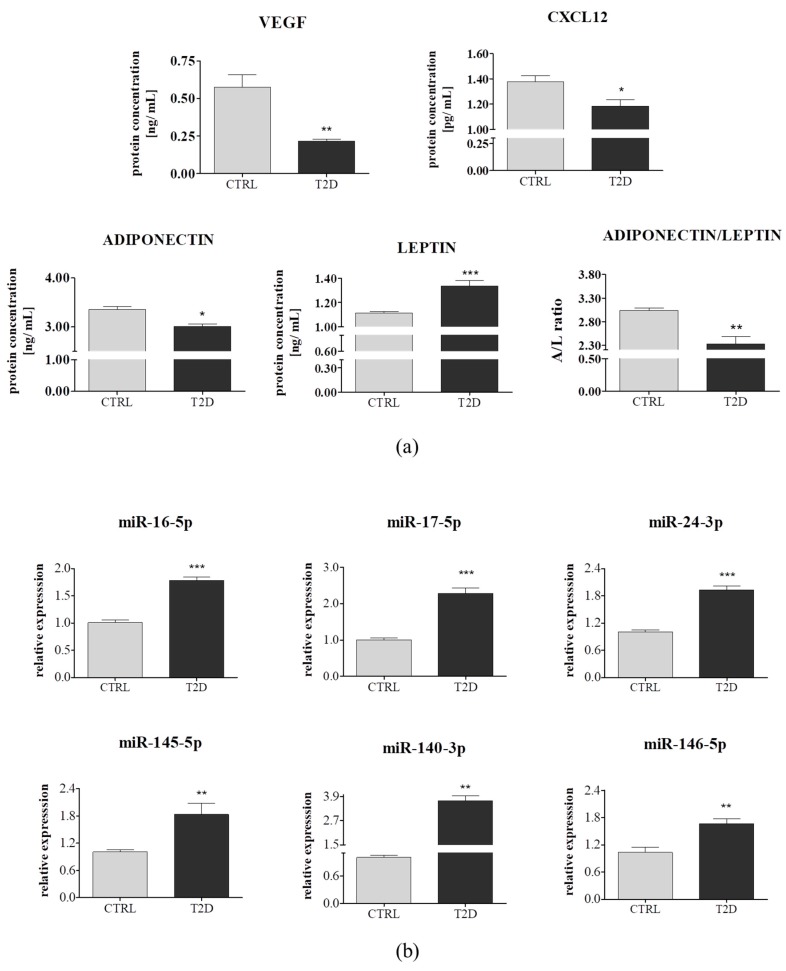
Comparison of selected miRNAs and proteins secreted by adipose-derived mesenchymal stem cells, isolated from non-diabetic (*n* = 6) individuals and patients suffering from type 2 diabetes (T2D) (*n* = 6). (**a**) Proteome analysis of the conditioned media from the ASC_CTRL_ and the ASC_T2D_ was performed using the ELISA method. (**b**) miRNA levels in extracellular vesicles extracted using Amicon Ultra 15 filter (3000 MWCO). Extracellular miRNA expression profiling was determined using quantitative PCR. Results expressed as mean ± SD. Statistical significance indicated as asterisk (*): * *p* < 0.05, ** *p* < 0.01, and *** *p* < 0.001 were examined through unpaired Student’s *t*-test.

The use of ASCs in regenerative medicine included cell-free therapies such as application of secretome, besides the stem cell transplantation. ASCs produce and secrete a wide array of chemokines, growth factors, and extracellular vesicles containing mRNA and miRNA. ASC secretome can prevent cell apoptosis, and promote endogenous repair mechanisms and angiogenesis. To evaluate and compare the concentration of VEGF, CXCL12, leptin, and total adiponectin/acrp30 in conditioned media collected from ASC_CTRL_ and ASC_T2D_, ELISA assays were performed. In the ASC_CTRL_ culture medium, we detected a significant higher concentration of VEGF (*p* > 0.01), that mediated the differentiation of endothelial progenitor cells and induced corneal neovascularization, in vivo [57]. It is well-documented that VEGF level is decreased in type 2 diabetic patients sera, which is directly linked to the impairment of angiogenesis in diabetic conditions [12,57]. Moreover, we observed a downregulation of CXCL12 (also named stromal cell-derived factor-1, SDF-1) (*p* < 0.05) and adiponectin (*p* < 0.01), whereas an overexpression of leptin (*p* < 0.001) significantly reduced the adiponectin/leptin ratio (*p* < 0.01) (Figure 5A). CXCL12 plays a role in diverse cellular functions, including response to inflammation and maintaining the stemness of hematopoietic stem cells (HSCs). Hu et al. previously reported that CXCL12 accelerated the epithelialization of burn wound through MSC mobilization [58]. Our result was consistent with the findings of Kočí et al. who showed that ASC_T2D_ were typically prone to downregulated CXCL12 [59]. Leptin and adiponectin had opposite effects on insulin resistance and chronic inflammation. Leptin upregulated pro-inflammatory cytokines, whereas adiponectin exhibited anti-inflammatory properties. The observed adiponectin/leptin imbalance suggested that ASCs derived from diabetic patients exhibit impaired immunomodulatory properties.

Extracellular vesicles, including MVs and exosomes were extracted with an ultracel centrifugal filter device, with a 3 kDa molecular weight cut-off (MWCO). Isolated EVs were subsequently subjected to an extracellular miRNAs level analysis, using qRT-PCR. Interestingly, we observed a similar correlation between the intracellular and extracellular miRNAs expression (Figure 5B), suggesting that the expression level of the circulating miRNAs, probably represented a balance between miRNA release and uptake.

## 4. Conclusions

ASCs are considered to be a highly attractive therapeutic strategy, whereas, their physiological deterioration in the diabetic condition, limits their use in autologous transplantation. The obtained results confirmed that type 2 diabetes impairs critical functions of ASCs, such as viability, proliferation activity, mitochondrial dynamics, protective mechanisms against oxidative stress, as well as secretory capacity. Moreover, our findings suggest that metabolic disorders lead to an impaired glucose homeostasis and insulin sensitivity, through the downregulation of several mRNA and miRNA, which play an important role in glucose and lipid metabolism. Thus, it seems that before any clinical applications of ASCs derived from diabetic patient can be implemented, their pharmacological ex vivo improvement is strongly required.

## Figures and Tables

**Table 1:** List of genes with accession numbers, sequences of primers, and the amplicon size of the products in quantitative RT-PCR.

Gene	Primer Sequence (Forward/Reverse)	Amplicon Size (bp)	Accession Number
**SIRT-1**	ACAGGTTGCGGGAATCCAAA/	155	NM_001314049.1
	GTTCATCAGCTGGGCACCTA		
**HIF-1α**	TTCCTTCTCTTCTCCGCGTG/	175	NM_1810542
	TGGCTGCATCTCGAGACTTTT		
**LIN28**	CCGAACCCCATGCGCACGTT/	137	XM_011542148.2
	TTTGCAGGTGGCTGCGCCAAG		
**TGFβ**	GTTCTTCAATGCGTCGGAGC/	214	NM_001081849.1
	CACGACTCCGGTGACATCAA		
**p21**	AGAAGAGGCTGGTGGCTATTT/	169	NM_001220777.1
	CCCGCCATTAGCGCATCAC		
**p53**	AGATAGCGATGGTCTGGC/	381	NM_001126118.1
	TTGGGCAGTGCTCGCTTAGT		
**BAX**	ACCAAGAAGCTGAGCGAGTGTC/	365	XM_011527191.1
	ACAAAGATGGTCACGGTCTGCC		
**Cas-3**	GCGGTTGTAGAAGTTAATAAAGGT/	232	NM_001354784.1
	CGACATCTGTACCAGACCGAG		
**TERT**	CGGAAGAGTGTCTGGAGCAA	107	XM_011514106.1
	GGATGAAGCGGAGTCTGGA		
**MFN1**	GTTGCCGGGTGATAGTTGGA/	270	XM_005247596.4
	TGCCACCTTCATGTGTCTCC		
**FIS1**	TGGTGCGGAGCAAGTACAAT/	251	NM_016068.2
	TGCCCACGAGTCCATCTTTC		
**PRKN**	GTGCAGAGACCGTGGAGAAA/	291	XM_017010909.2
	GCTGCACTGTACCCTGAGTT		
**FOXO1**	ATTGAGCGCTTGGACTGTGA	311	XM_014732057.1
	CGCTGCCAAGTTTGACGAAA		
**GAPDH**	GTCAGTGGTGGACCTGACCT/	256	NM_001289746.1
	CACCACCCTGTTGCTGTAGC		

SIRT-1—sirtuin1; HIF-1α—hypoxia inducible factor 1 alpha subunit; LIN28A—lin-28 homolog A; TGF beta—transforming growth factor beta; p21—cyclin dependent kinase inhibitor 1A; p53—tumor suppressor p53; BAX—Bcl-2 associated X protein; Cas-3—Caspase-3; TERT—telomerase reverse transcriptase; MFN1—mitofusin 1; FIS1—mitochondrial fission 1 molecule; PRKN—Parkin; FOXO1—Forkhead Box O1; and GAPDH—Glyceraldehyde 3-phosphate dehydrogenase.

**Table 2:** Sequences of the primers used in the qPCR for the miRNA expression analysis.

Gene	Primer Sequence (5′→3′)	Accession Number
miR-24-3p	TGGCTCAGTTCAGCAGGAACAG	MIMAT0000080
miR-17-5p	CAAAGTGCTTACAGTGCAGGTAG	MIMAT0000070
miR-16-5p	TAGCAGCACGTAAATATTGGCG	MIMAT0000069
miR-140-3p	TACCACAGGGTAGAACCACGGA	MIMAT0004597
miR-146a-5p	TGAGAACTGAATTCCATGGGTT	MIMAT0000449
miR-145-5p	GTCCAGTTTTCCCAGGAATCCCT	MIMAT0000437
